# A nationwide cross-sectional study evaluating fertility knowledge, attitudes and parenting aspirations among undergraduate medical students in Pakistan

**DOI:** 10.1097/MS9.0000000000003123

**Published:** 2025-03-07

**Authors:** Abdul Moiz Khan, Syeda Shahnoor, Adeena Maryyum, Syed Hassan Ahmed, Muhammad Khubaib Ullah, Sawsane Ghaddar, Maryam Irshad, Sania Ghaffar, Aman Goyal

**Affiliations:** a Department of Internal Medicine, Ayub Medical College, Abbottabad, Pakistan; b Department of Internal Medicine, Dow University Of Health Sciences, Karachi, Pakistan; c Faculty of Medical Sciences, Lebanese University, Beirut, Lebanon; d Department of Internal Medicine, King Edward Memorial Hospital, Mumbai, India

**Keywords:** age-related fertility decline, fertility awareness, infertility, medical students, parenthood

## Abstract

**Introduction:**

According to the WHO, infertility affects 17.5% of the global population. The trend of delaying parenthood, especially among highly educated individuals, has contributed to reduced fertility and a higher risk of pregnancy complications. Medical students, due to prolonged education and demanding careers, are at a higher risk of struggling to have children. Despite this, little research has been conducted on their fertility awareness. This study aims to assess fertility knowledge, attitudes, and parenting aspirations – such as goals and plans for future parenthood – among medical students across Pakistan, while also addressing the often-overlooked aspect of male fertility awareness, including factors like age-related fertility decline.

**Materials and methods:**

This descriptive cross-sectional study was conducted from July to December 2023, targeting medical students from various colleges across Pakistan. A sample size of 383 participants was determined using Raosoft software. Data was collected via an online questionnaire adapted from the validated Swedish Fertility Awareness Questionnaire.

**Results:**

The study included 384 participants from 63 medical institutes across Pakistan, comprising 145 men and 239 women enrolled in undergraduate medical programs. A high proportion of participants (93.1% males, 90.8% females) intend to have children, with 31% of men and 39.3% of women planning for three children. Approximately one-third (33.1%) of male participants preferred having their last child between 35 to 39 years, while about half (53.6%) of women preferred having their last child between 30 to 34 years. Many participants (98.6% men, 97.9% women) incorrectly perceive the age at which female fertility begins to decline as being later than it actually is. Knowledge about assisted reproductive technologies is limited, with only 10.3% of males and 20.5% of females understanding in-vitro fertilization. Factors influencing decisions to have children include partner support, stable relationships, and financial security.

**Conclusion:**

This study sheds light on fertility knowledge and aspirations among Pakistani medical students, revealing strong desires for parenthood and larger families. However, it exposes misconceptions about fertility and highlights the need for improved education and access to fertility services.

## Introduction

Infertility is a medical condition characterized by the inability to conceive after engaging in regular unprotected sexual intercourse for a duration of 12 months or longer ^[^1^]^. A 2023 report by WHO reveals that approximately 17.5% of the global adult population (1 in 6 individuals) experience infertility issues ^[^2^]^. Infertility can stem from hormonal imbalances, structural problems in reproductive organs, infections, lifestyle choices like smoking and excessive alcohol, genetics, as well as age-related decline and postponement of parenthood^[^3^]^. Delaying childbirth has become increasingly common in industrialized countries, particularly among highly educated individuals, who often wait until they achieve stability in education, career, finances, and housing^[^4^]^. However, this choice has consequences, such as reduced fertility and increased risks during pregnancy and childbirth, including complications for both mother and fetus like ectopic pregnancies, miscarriages, premature labor, and genetic and congenital birth defects^[^5^]^. Men also experience a notable decline in fertility as they age, with detectable changes occurring after 35 years and this decrease continues to worsen beyond the age of 50^[^6^]^.

**Highlights**

- The research assessed fertility knowledge, attitudes, and parenting aspirations among 384 medical students in Pakistan, including male fertility awareness.
- Participants overestimated female fertility decline age and had limited understanding of assisted reproductive technologies like in-vitro fertilization.
- Most students plan to have children, prefer larger families, and have specific ideal ages for childbearing, influenced by partner support, stability, and financial security.
- Findings emphasize the need for enhanced fertility education and access to reproductive health services, with recommendations for future research on socio-cultural factors.

In light of the global decline in fertility rates and the increasing prevalence of infertility, understanding the level of fertility knowledge among individuals becomes crucial. Several international studies assessing fertility awareness have revealed limited knowledge regarding infertility risk factors and fertility topics, among university students, often accompanied by overly optimistic views on natural conception and the success of Assisted Reproductive Technology (ART)^[^7-10^]^. In Pakistan, educated women are experiencing a rising trend in the median maternal age at first birth, indicating that current higher education students in the country are likely to further delay parenthood^[^11^]^. These trends are even more common in medical students^[^12^]^. Medical professionals, particularly, face fertility and family planning challenges due to demanding careers and extended education that coincide with their prime reproductive years^[^13^]^. In Pakistan, medical education spans 5 years, followed by 4–5 years of fellowship training, increasing the risk of involuntary childlessness for medical students and trainees compared to other professionals.

Conducting a nationwide study concerning the issue of infertility in Pakistan holds immense significance owing to its status as the fifth most populous country in the world^[^14^]^. Our study aims to assess medical student’s current understanding of reproductive health, fertility treatments, and family planning, while also exploring their perspective on parenthood, including family size preferences, ideal age to start a family, and the societal and cultural factors shaping their views on parenting and fertility. This pioneering survey captures the diverse views of medical students across the entire country which is impertinent for developing comprehensive and region-specific reproductive health programs and curricula, ultimately fostering more efficient healthcare practices.

## Materials and methods

### Ethics statement

Prior to the commencement of this study, strict adherence to ethical protocols was ensured. Ethical approval was obtained from the institutional review board (EA-2023/091). The investigation was performed with complete adherence to the principles of the Helsinki Declaration^[^15^]^. The study was carried in accordance with the reporting guidelines specified by the STROCSS criteria^[^16^]^. Informed consent was obtained from all study participants before any data procurement took place.

### Study design, setting, and participants

The research was a descriptive cross-sectional study conducted from July to December 2023. The study included medical students from medical colleges across Pakistan, representing both government and private sectors. Inclusion criteria comprised medical students enrolled in a Pakistan Medical and Dental Council (PMDC) recognized undergraduate medical program within Pakistan. Notably, students pursuing healthcare programs other than MBBS were deliberately excluded from the study.

The sample size was calculated using an online sample size calculator, Raosoft software. The estimated population size of undergraduate medical students in Pakistan, as reported by The Nation newspaper, stood around 75 000^[^17^]^. A confidence level of 95%, an absolute precision of 5%, and a response distribution of 50% gave us a sample size of 383 participants.

To gather data concerning fertility knowledge and attitudes among medical students, we adapted Akhtar H’s adaptation of the validated Swedish Fertility Awareness Questionnaire^[^11^]^, originally developed by Lampic *et al*^[^18^]^. Necessary modifications were thoughtfully made to align the questionnaire with the characteristics of the study population. Given the questionnaire’s established utilization in previous studies, pilot testing was deemed redundant. The sample was recruited using a nonprobability convenience sampling technique, ensuring diversity among participants. The data collection process involved the administration of an online questionnaire, distributed through various social platforms.

### Data analysis

The collected data was analyzed using SPSS version 23. Categorical data were statistically analyzed using the Chi-square test, while the means of continuous and categorical data were subjected to independent sample *t*-test. Statistical scrutiny was conducted with a 95% confidence interval, and a *P*-value below 0.05 was deemed statistically significant.

## Results

This nationwide cross-sectional study included 384 participants from 63 medical institutes, comprising of 145 men and 239 women. Over half of the participants resided in Khyber Pakhtunkhwa (56.3%), followed by Punjab (24.2%) and Sindh (9.9%) respectively. The remaining 9.6% of medical students resided in Baluchistan, Azad Jammu Kashmir, and Islamabad. The mean age among male participants was observed to be 21.70 ± 1.879 years, in contrast to 21.40 ± 1.657 years among female medical students. Almost one-third of participants (37.2%) had studied gynecology and obstetrics as a part of their medical curriculum, with 40% men and 35.6% women. Moreover, 26% of women reported being in a stable relationship as compared to 22% men. The baseline characteristics of the participants included have been summarized in Table 1.

**Table 1:** Baseline characteristics of included participants

Baseline characteristics	Men (n = 145)	Women (239)
Age, years (mean ± SD)	21.70 ± 1.879	21.40 ± 1.657
**Province of residence**		
AJK	1 (0.7)	7 (2.9)
Baluchistan	7 (4.8)	0 (0.0)
Islamabad Capital Territory	6 (4.1)	16 (6.7)
KPK	100 (69.0)	116 (48.5)
Punjab	19 (13.1)	74 (31.0)
Sindh	12 (8.3)	26 (10.9)
**Year of study**		
1st Year	32 (22.1)	41 (17.2)
2nd Year	39 (26.9)	44 (18.4)
3rd Year	28 (19.3)	63 (26.4)
4th Year	18 (12.4)	49 (20.5)
5th Year	28 (19.3)	42 (17.2)
**Studied gynecology/obstetrics**		
Yes	58 (40.0)	85 (35.6)
No	87 (60.0)	154 (64.4)
**Are you in a stable relationship?**		
Yes	22 (15.2)	26 (10.9)
No	123 (84.8)	213 (89.1)

When asked about how important it was for them to have children on a scale of 0 (not important) to 10 (very important), a mean score of 7.7 ± 2.794 and 7.41 ± 2.927 was observed among men and women respectively. About 93.1% male and 90.8% female participants respectively planned to have children in the future, with 31% of men and 39.3% of women planning to have 3 children. Additionally, 13.1% men and 3.3% women showed intentions of having 5 or more children. Furthermore, 46.2% men and 70.3% women preferred to have their first child between 25 and 28 years of age. Similarly, 33.1% male participants preferred having their last child between 35 and 39 years, whereas 53.6% women preferred having their last child between 30 and 34 years. The results have been tabulated in Tables 2 and 3.

**Table 2:** Intention of having children among participants

Items	Men (n = 145)	Women (n = 239)
**Do you plan to have children in future?**		
Yes	135 (93.1)	217 (90.8)
No	10 (6.9)	22 (9.2)
**How many children do you want?**		
1	4 (2.7)	8 (3.3)
2	42 (29.0)	80 (33.5)
3	45 (31.0)	94 (39.3)
4	25 (17.2)	27 (11.3)
5	8 (5.5)	4 (1.7)
6	6 (4.1)	2 (0.8)
7	1 (0.7)	0 (0.0)
8	0 (0)	0 (0.0)
9	0 (0)	1 (0.4)
10	4 (2.8)	1 (0.4)
I do not want a child	10 (6.9)	22 (9.2)
**At what age would you like to have your first child?**		
18–20	0 (0.0)	2 (0.8)
21–24	6 (4.1)	6 (2.5)
25–28	80 (46.2)	163 (70.3)
29–32	44 (38.0)	41 (17.1)
33–36	5 (3.4)	0 (0.0)
I do not want a child	10 (6.9)	22 (9.2)
**At what age would you like to have your last child?**		
24–29	9 (6.2)	19 (7.8)
30–34	44 (30.3)	128 (53.6)
35–39	48 (33.1)	63 (26.4)
40–44	26 (17.9)	5 (2.9)
45–50	8 (5.5)	2 (0.8)
I do not want a child	10 (6.9)	22 (9.2)

**Table 3:** Importance of having children

Item	Sex	Mean	Std. deviation	*P*-value
**How important is it for you to have children?**	Men	7.77	2.794	0.236
	Women	7.41	2.927	
**What would you do if you and your partner couldn’t conceive naturally?**				
We would opt for in vitro fertilization (IVF)	M	4.19	3.662	0.002
	F	5.42	3.654	
We would adopt a child	M	4.03	3.252	0.116
	F	4.57	3.250	
We would choose not to have children	M	2.95	3.363	0.046
	F	3.66	3.332	

When assessing participants’ knowledge about fertility, 60.7% men and 57.7% women correctly estimated the age at which women demonstrated the highest fertility, with the rest either underestimating or overestimating. 98.6% men and 97.9% women overestimated the age at which females experience a slight decline in their ability to get pregnant. Similarly, only 11% men and 13.4% women correctly identified the age at which a marked reduction is observed. Inadequate knowledge was observed among participants when inquired about chances of getting a women pregnant after having unprotected intercourse, with only 9% men and 4.6% women identifying the correct option, in women less than 25 years. Among higher age groups, comparatively more participants were able to correctly identify the correct chances of getting pregnant, as shown in Table 4. Similarly, inadequate knowledge was observed about male fertility, with the majority unable to recognize the correct options. Moreover, only 10.3% male and 20.5% female had adequate knowledge about in-vitro fertilization to enable them to select the right option when asked about chances of getting a child after one treatment.

**Table 4:** Participants awareness of fertility

Items	Men (n = 145)	Women (n = 239)	*P*-Value
**At what age are women most fertile?**			0.098
15–19 years	22 (15.2)	21 (8.8)	
20–24 years*	88 (60.7)	138 (57.7)	
25–29 years	34 (23.4)	76 (31.8)	
30–34 years	1 (0.7)	4 (1.7)	
**At what age is there a slight decrease in women’s ability to become pregnant?**			0.092
25–29 years*	2 (1.4)	5 (2.1)	
30–34 years	18 (12.4)	49 (20.5)	
35–39 years	53 (36.6)	98 (41.0)	
40–44 years	56 (38.6)	69.0 (28.9)	
45–59 years	16 (11.0)	18 (7.5)	
**At what age is there a marked decrease in women’s ability to become pregnant?**			0.713
25-29 years	0 (0.0)	1 (0.4)	
30–34 years	4 (2.8)	5 (2.1)	
35–39 years*	16 (11.0)	32 (13.4)	
40–44 years	53 (36.6)	96 (40.2)	
45–59 years	72 (49.7)	105 (43.2)	
**A young woman (<25 years) and a man have unprotected intercourse at the time of ovulation: What is the likelihood of her becoming pregnant?**			0.070
0–29 %	12 (8.3)	5 (2.1)	
30–39 %*	13 (9.0)	11 (4.6)	
40–49 %	21 (14.5)	27 (11.3)	
50–59 %	23 (15.9)	53 (22.2)	
60–100 %	76 (52.4)	143 (59.8)	
**A woman and a man have had regular unprotected intercourse over a period of 1 year: What is the likelihood of the woman becoming pregnant, if she is 25-30 years old?**			0.109
0-20%	4 (2.8)	2 (0.8)	
21-40%	9 (6.2)	7 (2.9)	
41-60%	14 (9.7)	26 (10.9)	
61-80%*	58 (40.0)	120 (50.2)	
81-100%	60 (41.4)	84 (35.1)	
**A woman and a man have had regular unprotected intercourse over a period of 1 year: What is the likelihood of the woman becoming pregnant, if she is 35-40 years old?**			0.207
0–20%	11 (7.6)	17 (7.1)	
21–40%	32 (22.1)	69 (28.9)	
41–60%*	56 (38.6)	102 (42.7)	
61–80%	37 (25.5)	40 (16.7)	
81–100%	9 (6.2)	11 (4.6)	
**At what age are men most fertile?**			<0.001
15–19 years	13 (9.0)	6 (2.5)	
20–24 years	57 (39.3)	62 (25.9)	
25–29 years	65 (44.8)	137 (57.3)	
30–34 years*	10 (6.9)	31 (13.0)	
35-44 years	0 (0.0)	3 (1.3)	
**At what age is there a slight decrease in men’s ability to impregnate a woman?**			0.095
25–29 years	3 (2.1)	3 (1.3)	
30–34 years	5 (3.4)	3 (1.3)	
35–39 years*	12 (8.3)	25 (10.5)	
40–44 years	37 (25.5)	87 (36.4)	
45–59 years	88 (60.7)	121 (50.6)	
**At what age is there a marked decrease in men’s ability to impregnate a woman?**			0.214
25–29 years	0 (0)	1 (0.4)	
30–34 years	3 (2.1)	0 (0.0)	
35–39 years	4 (2.8)	5 (2.1)	
40–44 years*	14 (9.7)	25 (10.5)	
45–59 years	124 (85.5)	208 (87)	
**If a couple undergoes treatment with in vitro fertilization (IVF)** – **what is their chance, on average, of getting a child after one treatment?**			<0.001
0–19%	9 (6.2)	25 (10.5)	
20–29%*	15 (10.3)	49 (20.5)	
30–39%	45 (31.0)	84 (35.1)	
40–49%	36 (24.8)	61 (25.5)	
50–100%	40 (27.6)	20 (8.4)	

*Indicates the correct answer.

When inquired about factors that could influence participants’ decision to have children, 66.2% men and 85.4% women considered having a partner they can share responsibilities with very important. Similarly, being in a stable relationship and having a good financial standing were also considered very important by the majority of participants across both genders, as shown in Table 5. A significantly higher proportion of female participants considered access to childcare, being able to manage work alongside more important than men, and feeling enough mature.

**Table 5:** Factors impacting participants decision to have children

Items	Men (n = 145)	Women (n = 239)	*P* value
**That I have a partner with whom I can share the responsibility**			0.001
No opinion	9 (6.2)	7 (2.9)	
Not very important	6 (4.1)	6 (2.5)	
Somewhat important	31 (21.4)	20 (8.4)	
Unimportant	3 (2.1)	2 (0.8)	
Very Important	96 (66.2)	204 (85.4)	
**That I feel sufficiently mature**			0.033
No opinion	8 (5.5)	9 (3.8)	
Not very important	10 (6.9)	7 (2.9)	
Somewhat important	38 (26.2)	51 (21.3)	
Unimportant	5 (3.4)	2 (0.8)	
Very Important	84 (57.9)	170 (71.1)	
**That I want to have children before I am “too old”**			0.440
No opinion	6 (4.1)	7 (2.9)	
Not very important	12 (8.3)	23 (9.6)	
Somewhat important	47 (32.4)	91 (38.1)	
Unimportant	8 (5.5)	6 (2.5)	
Very Important	72 (49.7)	112 (46.9)	
**That I am in a stable relationship**			0.003
No opinion	6 (4.1)	5 (2.1)	
Not very important	11 (7.6)	5 (2.1)	
Somewhat important	17 (11.7)	16 (6.7)	
Unimportant	5 (3.4)	2 (0.8)	
Very Important	106 (73.1)	211 (88.3)	
**That my work can be combined with having children**			<0.001
No opinion	14 (9.7)	11 (4.6)	
Not very important	23 (15.9)	15 (6.3)	
Somewhat important	55 (37.9)	68 (28.5)	
Unimportant	8 (5.5)	6 (2.5)	
Very Important	45 (31.0)	139 (58.2)	
**That I have completed my studies**			0.002
No opinion	11 (7.6)	6 (2.5)	
Not very important	25 (17.2)	42 (17.6)	
Somewhat important	31 (21.4)	55 (23.0)	
Unimportant	10 (6.9)	2 (0.8)	
Very Important	68 (46.9)	134 (56.1)	
**That I have advanced in my profession**			0.096
No opinion	10 (6.9)	9 (3.8)	
Not very important	21 (14.5)	42 (17.6)	
Somewhat important	52 (35.9)	92 (38.5)	
Unimportant	11 (7.6)	6 (2.5)	
Very Important	51 (35.2)	90 (37.7)	
**That I have access to childcare**			0.001
No opinion	9 (6.2)	10 (4.2)	
Not very important	13 (9.0)	17 (7.1)	
Somewhat important	48 (33.1)	52 (21.8)	
Unimportant	10 (6.9)	4 (1.7)	
Very Important	65 (44.8)	156 (65.3)	
**That I am/we are financially stable**			<0.001
No opinion	6 (4.1)	7 (2.9)	
Not very important	12 (8.3)	4 (1.7)	
Somewhat important	31 (21.4)	40 (16.7)	
Unimportant	8 (5.5)	3 (1.3)	
Very Important	88 (60.7)	185 (77.4)	

The expectations from parenthood also varied among the two genders with the majority among both men and women expecting development as a person alongside giving and receiving more love. Significant differences were observed between the two groups when less time to devote to career, less time for own interests, more close contact with family, a more enjoyable life, and feeling complete were evaluated. The responses have been tabulated in Table 6. Lastly, age, genetics, smoking, drugs, and BMI were regarded as factors negatively impacting fertility by the majority of participants, as shown in Table 7.

**Table 6:** Participants opinion on parenthood

Items	Men (n = 145)	Women (n = 239)	P-value
**I will develop as a person**			0.305
Disagree	4 (2.8)	9 (3.8)	
Moderately agree	30 (20.7)	69 (28.9)	
No opinion	4 (2.8)	7 (2.9)	
Somewhat agree	9 (6.2)	19 (7.9)	
Strongly agree	98 67.6)	135 (56.5)	
**I will have less time to devote to work and a career**			<0.001
Disagree	42 (29.0)	24 (10.0)	
Moderately agree	45 (31.0)	94 (39.3)	
No opinion	7 (4.8)	8 (3.3)	
Somewhat agree	35 (24.1)	66 (27.6)	
Strongly agree	16 (11.0)	47 (19.7)	
**I will give and receive more love**			0.868
Disagree	5 (3.4)	8 (3.3)	
Moderately agree	35 (24.1)	58 (24.3)	
No opinion	3 (2.1)	10 (4.2)	
Somewhat agree	14 (9.7)	22 (9.2)	
Strongly agree	88 (60.7)	141 (59.0)	
**I will have less time for my own interests**			0.014
Disagree	29 (20.0)	25 (10.5)	
Moderately agree	52 (35.9)	81 (33.9)	
No opinion	5 (3.4)	7 (2.9)	
Somewhat agree	36 (24.8)	57 (23.8)	
Strongly agree	23 (15.9)	69 (28.9)	
**I will have more contact with my close family**			<0.001
Disagree	9 (6.2)	20 (8.4)	
Moderately agree	42 (29.0)	97 (40.6)	
No opinion	5 (3.4)	21 (8.8)	
Somewhat agree	17 (11.7)	41 (17.2)	
Strongly agree	72 (49.7)	60 (25.1)	
**I will have less freedom**			0.231
Disagree	45 (31.0)	55 (23.0)	
Moderately agree	40 (27.6)	74 (31.0)	
No opinion	8 (5.5)	12 (5.0)	
Somewhat agree	34 (23.4)	51 (21.3)	
Strongly agree	18 (12.4)	47 (19.7)	
**My life will become more enjoyable**			0.012
Disagree	9 (6.2)	25 (10.5)	
Moderately agree	48 (33.1)	79 (33.1)	
No opinion	9 (6.2)	17 (7.1)	
Somewhat agree	15 (10.3)	48 (20.1)	
Strongly agree	64 (44.1)	70 (29.3)	
**I will have less financial stability**			0.287
Disagree	72 (49.7)	108 (45.2)	
Moderately agree	29 (20.0)	49 (20.5)	
No opinion	7 (4.8)	22 (9.2)	
Somewhat agree	26 (17.9)	50 (20.9)	
Strongly agree	11 (7.6)	10 (4.2)	
**I will feel complete**			0.005
Disagree	22 (15.2)	63 (26.4)	
Moderately agree	40 (27.6)	45 (18.8)	
No opinion	5 (3.4)	20 (8.4)	
Somewhat agree	14 (9.7)	30 (12.6)	
Strongly agree	64 (44.1)	81 (33.9)	
**Will have strain on my relationship with my partner**			0.57
Disagree	64 (44.1)	121 (50.6)	
Moderately agree	25 (17.2)	32 (13.4)	
No opinion	20 (13.8)	38 (15.9)	
Somewhat agree	24 (16.6)	31 (13.0)	
Strongly agree	12 (8.3)	17 (7.1)

**Table 7:** Factors negatively impacting fertility

Factors	Men (n = 145)	Women (n = 239)
Age	113 (77.9)	201 (84.1)
Genetics	98 (67.6)	186 (77.8)
Sleep	50 (34.5)	63 (26.4)
Smoking	111 (76.6)	192 (80.3)
Drugs	122 (84.1)	214 (89.5)
Diet	78 (53.8)	132 (55.2)
Sedentarism	66 (45.5)	102 (42.7)
BMI	76 (52.4)	150 (62.8)

## Discussion

This study represents the first nationwide investigation across Pakistan into fertility awareness among medical students, particularly age-related decline, along with an exploration into their attitudes and intentions regarding parenthood. Our findings underscore a prevalent positive attitude towards parenthood among the majority (90%) of participating students, revealing a strong desire to have children in the future. Notably, a significant proportion of both male and female participants expressed a preference for three children, a figure closely aligned with Pakistan’s fertility rate of 3.2 in 2023^[^19^]^. This contrasts with prior studies conducted in Karachi^[^11^]^, Iraq^[^20^]^, and Egypt^[^21^]^ where medical students indicated intentions to have fewer children than the general population of their respective countries. However, it is pertinent to note that Pakistan ideally needs to achieve a population growth rate of 2.1 to ensure sustainable development^[^22^]^. This high fertility intentions observed among medical students in our study may stem from factors such as higher earning potential, financial stability, and positive relationships within the medical community, which could enable them to support larger families^[^23,24^]^

Moreover, our findings indicate a preference among both male and female participants to delay childbirth to later ages, with a notable proportion aiming to have their last child after the age of 35. This trend mirrors similar findings from studies conducted among medical students in other countries^[^7,10,20^]^. However, this inclination towards delayed childbearing raises concerns, as advanced maternal and paternal age are associated with increased childbirth complications^[^25^]^. Our analysis reveals that a substantial majority of participants overestimated the age at which women experience declines in fertility, highlighting widespread misconceptions that may contribute to delayed childbearing and associated health risks. This lack of accurate knowledge may partly explain the prevalence of childbirth complications in Pakistan^[^26,27^]^. Pakistan stands as the second-highest nation globally in terms of stillbirth rates^[^28^]^, and is third on the list of countries with highest neonatal mortality rates^[^29^]^. Indeed, advanced maternal age emerges as a significant contributor to childbirth complications such as neonatal mortality^[^30^]^.

Moreover, participants demonstrated unrealistic expectations regarding the likelihood of conception after unprotected intercourse. This overestimation can lead to undue stress and frustration for couples experiencing difficulties conceiving, often resulting in undue blame placed on the female partner^[^31,32^]^. Addressing these misconceptions is crucial for promoting informed decision-making and reducing the stigma surrounding infertility^[^33^]^. Additionally, our findings underscore a notable disparity in knowledge concerning male fertility, with limited awareness of age-related declines in sperm quality and quantity. The prevailing belief that fertility issues primarily stem from female factors perpetuates the misconception that men’s fertility remains unaffected by age^[^34^]^ This highlights the urgent need for comprehensive education initiatives to dispel myths and misconceptions surrounding male fertility and its impact on conception.

The study highlighted a significant lack of knowledge about IVF among participants, with few possessing sufficient understanding of the treatment process. Despite the observed lack of awareness, when participants were queried about their intended courses of action in the event of infertility, IVF emerged as the most preferred option, followed by adoption and choosing a child-free lifestyle. This preference aligns with similar studies conducted in other regions, such as Iraq, Serbia, and Sweden, where IVF was also rated as the primary choice in cases of infertility^[^10,18,20^]^. However, the prevalent interest in IVF highlights the importance of addressing the knowledge gap through enhanced educational initiatives and improved accessibility to IVF-related services. By providing individuals with accurate information about fertility treatments, including IVF, they can make more informed decisions regarding their reproductive options.

In our study, adopting a child in the event of childlessness emerged as the second most favored option. These findings are comparable to those from Mexico, where adoption was identified as the primary choice^[^8^]^. Intra-family adoptions in case of childlessness are common practice conducted across various countries culturally and religiously^[^35,36^]^. This reflects a willingness within these societies to explore non-conventional avenues for family-building, beyond traditional medical interventions. A study conducted in Karachi revealed that approximately 50% of women had considered adoption during their period of childlessness^[^37^]^. Despite the majority of these women (72.5%) receiving support from their husbands, there was notable reluctance from in-laws and relatives to pursue this option. Given that adoption presents a viable solution for individuals facing childlessness, it becomes crucial to offer counseling not only to the couple but also to their family members, including the in-laws, in instances where the underlying condition cannot be remedied^[^38^]^

Our study highlighted the crucial role of partnership dynamics in parenthood decisions, with most participants prioritizing having a supportive partner for sharing responsibilities. This emphasizes the importance of mutual support in raising children^[^39^]^

Furthermore, our findings emphasize the importance of having a healthy relationship with one’s partner and financial stability in determining readiness for parenthood. Top of form in Pakistan’s demanding economic environment, widespread financial strain presents formidable barriers to fulfilling parental duties. Families often face tough decisions, compromising child care and healthcare access for their children. This accentuates the essential role of financial stability amid broader socio-economic challenges^[^40^]^ Female participants prioritized access to childcare and balancing work with parenting, reflecting the unique societal expectations and challenges faced by women as caregivers and professionals. Moreover, our study sheds light on participants’ perceptions of the expectations and rewards associated with parenthood. Both men and women expressed aspirations for personal growth and enhanced emotional fulfillment through the experience of raising children, aligning with broader notions of parenthood as a transformative and enriching journey^[^41^]^.

Despite its strengths, this study has limitations. Reliance on self-reported data may be subject to response bias, potentially influencing the accuracy of participants’ knowledge assessments and attitudes toward fertility. The study’s cross-sectional design provides a snapshot at a specific point in time, limiting the assessment of longitudinal trends. Moreover, focusing primarily on medical students may not fully represent the broader population’s fertility perspectives and knowledge levels. Additionally, the use of an online questionnaire, while effective in reaching a diverse group of participants, may have excluded certain segments of the student population with limited internet access, potentially impacting the generalizability of the findings. Finally, while the study explores important aspects of fertility awareness and attitudes towards parenthood, a more comprehensive exploration of socio-cultural determinants, including religious beliefs, family dynamics, and societal norms, could provide a more nuanced understanding of fertility-related decision-making among medical students in Pakistan.

## Recommendations

To address the knowledge gaps, targeted educational initiatives should raise awareness about age-related fertility decline, male fertility, and IVF. Fertility education should be incorporated into university curricula, and public health campaigns should dispel fertility myths and promote realistic expectations. Additionally, counseling services for couples and their families, along with financial support programs and flexible work policies, can help mitigate socio-economic barriers to parenthood. Future studies should explore gender-based differences in fertility knowledge and attitudes with a focus on statistical significance, as this could provide deeper insights into tailored educational interventions

## Conclusion

This nationwide study sheds light on fertility knowledge, attitudes, and parenting aspirations among Pakistani medical students. The findings reveal a strong desire for parenthood, with preferences for larger families aligning closely with the country’s fertility rate. However, the study also highlights misconceptions and gaps in understanding regarding fertility and reproductive health, including age-related declines and conception likelihood. Interest in assisted reproductive technologies underscores the need for improved education and access to fertility services. Despite its strengths, the study’s reliance on self-reported data, cross-sectional design, and focus on medical students pose limitations in representing broader fertility perspectives. Future research should explore socio-cultural determinants to deepen understanding and inform tailored reproductive health programs and policies in Pakistan.

## Data Availability

The data used to support the findings of this study are available from the corresponding author upon request.
